# REMOTE MONITORING IN MULTISITE CLINICAL TRIALS: USING VIRTUAL TECHNOLOGY FOR RELIABILITY AND FIDELITY

**DOI:** 10.1093/geroni/igae098.0710

**Published:** 2024-12-31

**Authors:** Jennifer Stevens-Lapsley, Lauren Hinrichs-Kinney, Mattie Pontiff, Melissa Tran, Frances Westlake

**Affiliations:** University of Colorado Anschutz Medical Campus, Aurora, Colorado, United States; University of Colorado, Aurora, Colorado, United States; University of Colorado, Aurora, Colorado, United States; University of Colorado Anschutz Medical Campus, Aurora, Colorado, United States; University of Colorado, Aurora, Colorado, United States

## Abstract

Multi-site clinical studies often involve time consuming and resource intensive in-person monitoring of reliability and fidelity. An innovative solution provided by Swivl technologies holds tremendous potential to enhance remote monitoring options to reduce the need for costly in-person, onsite monitoring. The technology has enabled seamless and dynamic tracking of individuals undergoing Short Physical Performance Battery (SPPB) assessments, as well as specific rehabilitation interventions that are part of an ongoing 34 site clinical trial (NCT05492240) in skilled nursing facilities. The Swivl camera’s ability to video moving subjects while also using capturing conversations within a busy clinic environment uniquely enhances the quality of remote monitoring. Our team has used this technology to perform 190 SPPB reliability assessments and 65 rehabilitation fidelity assessments at 34 skilled nursing facility (SNF) clinics. This approach has allowed for real-time, remote observation of participants as they perform sit-to-stand, walking speed, and balance assessments within the SPPB. Similarly, the research team can remotely monitor fidelity of a high-intensity rehabilitation program focusing on dosing of specific rehabilitation exercises to ensure prescribed interventions are executed as intended. This virtual monitoring not only enhances the capacity to conduct remote reliability and fidelity assessments, but it also provides a valuable tool for continuous feedback and adjustment, optimizing the overall effectiveness of rehabilitation protocol fidelity. In essence, the Swivl camera brings forth a new era of remote monitoring that overcomes previous barriers (e.g. storage and transfer of large video files, inability to hear audio from an individual in a busy clinic setting).

